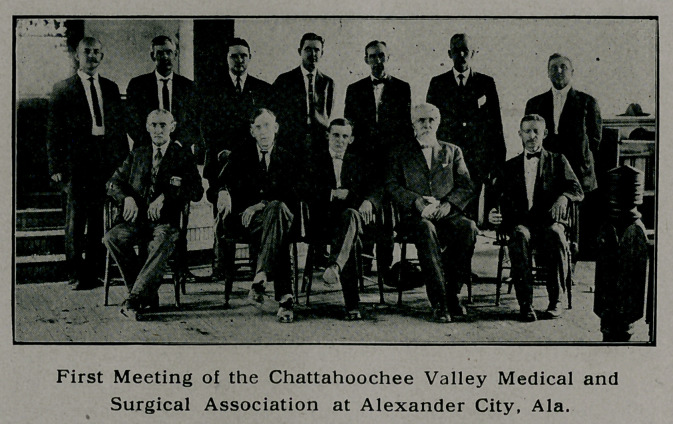# Personal Observations upon the Effects and Treatment of Chronic Constipation

**Published:** 1912-07

**Authors:** Lewis M. Gaines

**Affiliations:** Atlanta, Ga.; Professor of Neurology, Atlanta School of Medicine, Neurologist to Tabernacle Infirmary, Wesley Memorial Hospital and Georgian Hospital


### PERSONAL OBSERVATIONS UPON THE EFFECTS AND TREATMENT OF CHRONIC CONSTIPATION.

By Lewis M. Gaines, A. B., M .D., Atlanta, Ga.

*Professor of Neurology, Atlanta School of Medicine, Neurologist to Tabernacle Infirmary, Wesley Memorial Hospital and Georgian Hospital.*

I feel that I may be accused of possessing considerable temerity in venturing to present a paper upon such a subject. My conscience tells me that my distinguished friends, the gastro-enterologists, will accuse me of trespassing, while perhaps others will view with alarm the tedious prospect of listening to yet another presentation of a time-worn theme. In justification of my choice of subject, I desire to say that to the internist and neurologist the recognition of chronic constipation as a primary cause of a host of chronic ills masquerading under many misleading guises is of such importance that lack of recognition is suicidal to success in treatment. Indeed, I would say that all men everywhere who practice the healing art must recognize the role played by constipation in the causation of many major as well as minor ills if they are to meet with success in effecting the relief of these ills.

To be more specific, I will enumerate some of the conditions which in my judgment may have their roots in the prolonged absorption of the toxines resulting from retained fecal matter: arteriosclerosis, Bright's Disease, a host of functional disturbances of the nervous system usually grouped under the term neurasthenia, another host of kaleidoscopic, fugitive, elusive disturbances affecting the muscular and synovial tissues, usually grouped under the misleading term "rheumatism," another group of cases characterized by intellectual sluggishness, a feeling of general discomfort, offensiveness of the breath, furred tongue, with or without headache, vomiting of bile and abdominal pain. This group is most unfortunately designated by the very indefinite and unscientific term "biliousness." I have by no means exhausted the list of pathological conditions traceable to the toxemia resulting from infrequent or insufficient bowel evacuation; I have merely endeavored to indicate how extensive is the field of possibilities resulting from the neglected and often ill-treated condition.

I am guilty of no contradiction when I say that any of the diseased conditions I have just mentioned may be due to other causes than constipation. Thus arteriosclerosis or nephritis may result from the toxines of lead alcohol or syphilis. Again, what is called rheumatism may not be rheumatism at all, but the result of such a cause as a new growth or an aneurism with pressure symptoms. I wish to confess that I came to grief two years ago in just such a case. The patient complained of persistent pain and discomfort in the chest wall along the course of the intercostal nerves. There was a history of chronic constipation and a large excess of indican in the urine. Treatmnt was entirely unsuccessful. Physical examination gave no further clue to the cause, and I continued to be obessed by the idea that it was a toxic nuritis of intestinal origin. It was very disconcerting to find a few months later that signs of unmistakeable aneurism of the descending aorta has appeared. I had overlooked the possibility.

The reprehensible term "biliousness" may mean renal or hepatic colic, gall stones, cholecystitis or appendicitis. I wish to protest against such a term being used at all. Every time a physician uses the term in a life insurance examination it becomes necessary for the company to find out what is mean or not meant by it.

I may be justified in saying, then, that a correct diagnosis is extremely important and that before the source of the patient's ills are laid at the door of constipation, one must be sure they belong there.

Granting that any one of a large host of pathological conditons is due o chronic constipaton, which, by the way, may exist in the presence of daily evacuations, what shall we do for relief?

I have made it a point to question a large number of patients suffering from chronic constipation what they had been doing for the relief of the condition. Practcally without exception, all had become addicted to the continued use of more or less powerful drugs and were slaves to the enema. A great number took strong purgatives without which they declared a movement to be impossible. From purely personal observation I have concluded that although the treatment of chronic constipation is a hackneyed theme and that the majority of doctors feel that they know how to treat the condition, yet as a matter of fact rarely is the disturbance really cured, a disturbance fraught with so many serious possibilities.

The criteria of a cure are two:

(1) Complete regular evacuation of the bowels of normal amount and consistency.

(2) Freedom from continued dependence on drugs and mechanical appliances to secure such an evacuation.

I have treated a considerable number of patients for chronic constipation, and so far as I have information with uniform success. Nearly all of these patients came for the relief of some other condition which proved to be the result of their inefficient bowel action and resulting toxaemia.

Briefly stated, the method I have used while simple and by no means original with me consists in the following definite procedure:

(1) An initial thorough cleansing.

(2) An anti-constipation diet.

(3) Regular nightly ten-minute exercises for strengthening the abdominal muscles.

(4) Insistence upon habit formation in going to stool.

(5) Copious water drinking.

(6) The administration of agar with each meal and occasionally phenolphthalein at night.

(7) Lastly, but important, the regular administration of galvanism, faradism, or the high frequency current over the abdomen.

The time limit on this paper prevents me from going into details concerning the application of these procedures. I am convinced by my own experience that no case of constipation is too hopeless to respond to these methods, provided the patient will strictly follow direction and intelligently co-operate with the physician. The following case summaries will illustrate my contentions:

1. Miss L. B., aged 38, came to me for relief of neuralgic pains of three or four years' duration. For ten or fifteen years she has been a sufferer from chronic constipation. She had taken many different kinds of treatment with no benefit, so that she was entirely dependent upon purgatives and enemas for bowel movements. She was at once placed upon the regular treatment as outlined above and in three days her bowels were moving daily and abundantly without resort to drugs, although I continued her faradism for about three weeks, two or three times a week and insisted upon gradually diminishing amounts of agar. Agar can hardly be considered a drug as it is not at all absorbed, and acts entirely mechanically. The patient has remained free from constipation now for a long period and declars she is in better health than for years.

2. Mrs. F., aged 17, complained of pains in the back and abdomen, poor sleep and loss of appetite. She had suffered all her life from chronic constipation. She would frequently go for a week without a movement and occasionally two weeks would elapse between successive bowel movements. She was placed upon treatment and in an incredibly short time her bowels were moving daily with no drugs and her complaints disappeared.

In conclusnon, chronic constipation is an exceedingly common ailment. It is responsible for a great number of more or less serious diseased conditions. Its treatment is often inadequate and unsuccessful, judged by the criteria of complete regular evacuations without drugs. The methods outlined above have in my experience proven extremely satisfactory in practically every case and frequently a varied assortment of more or less disturbing complaints have cleared up and the patient restored to happiness as well as health.

1314 Empire Bldg.

---

**First Meeting of the Chattahoochee Valley Medical and Surgical Association at Alexander City, Ala.**

---

### SURGICAL SUGGESTIONS.

The history of a fairly sudden enlargement of a testicle does not necessarily mean an inflammatory or traumatic process. Such an enlargement may be due to spontaneous hemorrhage in a round-cell sarcoma of the organ.

Translucency in a scrotal swelling indicates, of course, the presence of hydrocele fluid But if the shadow of the testicle is unduly large the hydrocele is not only a complication of some other condition, e. g., neoplasm of the testis.—*American Journal of Surgery.*

## Figures and Tables

**Figure f1:**